# Cognitive Stimulation Programs in Healthy Elderly: A Review

**DOI:** 10.4061/2011/378934

**Published:** 2011-08-15

**Authors:** Sarah Tardif, Martine Simard

**Affiliations:** ^1^École de Psychologie, Université Laval Pavillon Félix-Antoine Savard, 2325 rue des Bibliothèques, local 1116, Québec, QC, Canada G1V 0A6; ^2^Centre de Recherche, Université Laval Robert-Giffard, Québec, QC, Canada G1J 2G3

## Abstract

This literature paper investigated the efficacy of 14 cognitive intervention programs administered to healthy elderly participants. PsycINFO and PubMed databases were searched using the following terms: cognitive training, cognitive stimulation, elderly, and aging. The majority of participants (13/14 studies) were recruited in community. Nine out of 14 studies targeted memory as the principal cognitive function to train or stimulate. Face-name associations, mental imagery, paired associations, and the method of loci were the main techniques taught to participants. Improvements were observed on at least one outcome measure in each study included in this paper. Recommendations to improve cognitive interventions in the healthy elderly are proposed, such as the utilization of more robust experimental designs, the inclusion of measures of generalization of training in daily life, the assessment of instrumental activities of daily living, quality of life, and self-esteem.

## 1. Introduction

In the current demographic context, aging and neurodegenerative diseases are well known and very much discussed in the media as they become a very important societal issue. Aging is usually related to decline and losses of various kinds. However, many elderly individuals want to remain physically and cognitively healthy. Individuals diagnosed with Alzheimer's disease (AD) have now access to pharmacological interventions that were developed to slow down and/or to stabilize the deterioration of cognitive functions. However, the available agents are only symptomatic treatments; there is no cure for AD. Three of the four available pharmacological agents target acetylcholine, which is known to play an important role in memory and is also known to be severely reduced in AD. In this sense, the most promising approach to date has been the development of cholinesterase inhibitors that facilitate cholinergic transmission. However, compliance to such treatments is limited by possible adverse effects [1]. Thus, the most promising avenues of intervention now lie in prevention. In this perspective, nutrition, physical activities, social interactions, and cognitive activities practiced by healthy elderly are currently the principal domains of interest. 

There are different types of nonpharmacological interventions. In cognitive intervention, the concepts of cognitive training, cognitive rehabilitation, and cognitive stimulation are the most popular approaches [2]. These approaches are complementary, and the choice of a particular approach depends on the objectives of the cognitive enhancement or maintenance and on the cognitive profile of the population targeted [3]. Cognitive training generally involves guided practice of standard tasks to increase or maintain particular cognitive functions such as memory [3, 4]. Cognitive rehabilitation, known as an individualized approach, also involves the practice of some tasks but generally targets personal goals in order to improve, one at a time, specific impairments in everyday life rather than improving performances on particular cognitive tasks [2–4]. The families are usually very much involved in cognitive rehabilitation in order to find strategies to reach the goals set for and/or by the patient [2–4]. Finally, cognitive stimulation promotes the involvement in activities that are aimed at a general enhancement of cognitive and social functioning, without specific objectives [2–4]. All three approaches can be useful for older adults with cognitive impairments while only cognitive training and stimulation are suitable for the healthy elderly. In the present paper, the concepts of stimulation/training programs will be used without distinction, because it is very difficult to concretely distinguish between stimulation and training programs and also because the selected studies frequently used both methods. These kinds of programs are hypothesized to impact on cognitive reserve, which is an important concept in aging. Cognitive reserve is generally known to delay the cognitive and functional expression of neurodegenerative diseases. In this sense, cognitive stimulation/training programs might have an impact on cognitive reserve, by optimizing normal performances, in agreement with the already known effect of education level [5, 6]. 

Other authors published literature reviews on cognitive intervention programs in the elderly in the past three years [3, 7–11]. However, these reviews analyzed cognitive interventions in both cognitively impaired and nonimpaired elderly participants [3, 7, 8, 11] which sometimes makes it more difficult to evaluate the specific impact of cognitive interventions in healthy elderly only. Some of these reviews also used a strict meta-analytic approach [9–11], and this approach is characterized by the utilization of very rigorous selection criteria that necessarily limit the number of reviewed studies. The objectives of the present paper were therefore to present the cognitive techniques used in cognitive training/stimulation programs, to review the results of the cognitive intervention programs administered to healthy elderly in the past ten years and up until March 2011, and to propose recommendations for future research.

## 2. Method

The terms *cognitive training, cognitive stimulation, elderly and aging *were searched in the PsycINFO and PubMed databases from January 2001 until March 2011. As a second step, the references of the articles found during the initial search were reviewed to identify any additional pertinent studies. Published articles were included if: (1) they were written in English or French; (2) the study involved at least a control group or condition, (3) the study used any type of cognitive training/stimulation among community dwelling healthy elderly, (4) the design included at least evaluations before and after intervention. Efficacy of the programs was ascertained in at least one of the two following ways: (1) significant results obtained following within-group comparisons involving evaluations before and after stimulation/training; and (2) significant results obtained following the comparisons between the trained and control groups after the intervention. Changes (i.e., improvement or deterioration) were considered significant in these two kinds of comparisons if *P* < .05.

## 3. Results

Fourteen studies met the inclusion criteria and were thus analyzed.  Table 1 presents the characteristics of the population investigated in the studies (participants' age, education, and gender), the sample size, study design, study duration, the cognitive functions targeted by the cognitive intervention, the type and form of cognitive stimulation/training, the outcome measures, and results of cognitive intervention. The studies are referenced according to their assigned number in Table 1. 

### 3.1. Design of the Studies

Nine studies were randomized-controlled studies (RC) [13, 15, 19, 21–25], a study was a controlled study [14], 2 were quasiexperimental [16, 17], and 2 studies used a within-subject crossover design [18, 20]. All studies included a control group, as per the inclusion criteria of the present paper. Seven studies used a nocontact or waiting-list group [13–16, 19, 21, 25]. Five studies had an active control group [12, 21–24] in order to obtain better comparisons. The studies using an active control group involved participants of this particular group in meetings with discussions [12] or in various activities in order to control for a specific stimulation effect of being part of a group or the capacity to use computers [23]. The principal activities proposed to the participants were some reading on diverse subjects [22] and watching DVD on literature and arts [21, 24]. It is important to mention that Mahncke et al. [21] and Slegers et al. [23] used more than one kind of control groups. Mahncke et al. used both an active and a passive control group in order to control for the effects of the active group. Slegers et al. trained a group to properly use computers without using it afterwards (active control group) and also involved in the study a group who did not receive any training and intervention as well as a group who had no interest in computers. Two studies used a within-subject crossover design that allowed good comparisons [18, 20]. Finally, the type of control group or control condition was not detailed in one study [17].

Out of the 14 studies listed in Table 1, 5 studies had no followup at all [14, 15, 19, 22, 24]. Nine studies had one or more followup evaluations after 60 months (1 study), 24 months (2 studies), 12 months (3 studies), 9 months (2 studies), 6 months (1 study), 4 months (1 study) and 3 months (3 studies) following the last cognitive intervention. Five studies had two followups at different times [12, 13, 16, 23, 25]. The mean number of training sessions was 26.91, (range from 3 [23] to 180 [16]). The duration of intervention sessions across the 13 studies was a mean of 1.60 hours (range from 1 to 4 hours). Nine studies administered group interventions, whereas 4 studies provided an individualized computer-based training [15, 21, 22, 24].

### 3.2. Recruitment Sites and Sociodemographics

Thirteen out of 14 studies recruited participants from the community, whereas two studies recruited participants from selected retirement homes [17, 25]. In addition, Willis et al. [25] included cognitively intact participants from community centers, hospitals, and clinics. Bherer et al. [15] also recruited young adults, and Belleville et al. [14] recruited patients with mild cognitive impairment (MCI) from memory clinics in order to compare their performances with those of healthy elderly. All participants of the 14 studies were healthy elderly, except for the patients with MCI involved in the study of Belleville et al. [14]. However, participants involved in 3 studies presented subjective memory complaints [16, 18, 20]. The mean age of participants involved in the 14 studies of this paper was 72.02 years. The mean years of education was 14.46 and 62% of the participants were women. 

### 3.3. Types of Interventions

**Cognitive Domains**

In 9 out of 14 studies the intervention mainly targeted training of memory [12–14, 16–19, 21, 25]. Most of the time, attention and executive functions were the other cognitive domains targeted by the interventions [15, 20, 22]. Speed of information-processing and general cognitive functioning were also trained and/or stimulated in some intervention programs [13, 21, 24, 25]. Executive functions are herein defined as the capacity of planning, organization, and reasoning. All these cognitive domains were evaluated before and after the interventions using several neuropsychological tests.

### 3.4. Tests

**Memory**

Multiple tests or subtests from a broader neuropsychological battery were used for baseline evaluations and as outcome measures. Verbal immediate and delayed recall of words, the Hopkins Verbal Learning Test [26], the Auditory Verbal Learning Test [27], the Rivermead Behavioral Memory Test (RBMT) [28], Logic Stories from the Wechsler Memory Scale-Revised (WMS-R) [29], other subtests from the WMS-III [30] as well as subtests from the Repeatable Battery for the Assessment of Neuropsychological Status (RBANS) [31] were the principal tests used as outcome measures for the efficacy evaluation.

**Attention**

A fewer number of tests were used to evaluate attentional functions compared with memory functions. Digit-span and Letter-Number sequences from the Weschler Adult Intelligence Scale-III (WAIS-III) [32] as well as the Brown-Petersen paradigm [33–35] were the tests mainly used to assess attention. Of interest, Bherer et al. [15] and Mozolic et al. [22] used an experimental computerized task especially designed for the purposes of their study.

**Executive Functions**

The Raven's matrices [36] nonverbal reasoning, completion of word and Letters series [37–39], verbal fluency [40], Simulated Real Life Tasks [41], Concept Shifting Test (authors' version of Trail Making Test) [42], Stroop Color-Word Test [43], Dysexecutive questionnaires [44], and the Clock Drawing Test [45] were used as executive measures (6 studies).

### 3.5. Techniques

All studies used different techniques or cognitive strategies in their specific interventions. The memory techniques taught to participants were face-name associations (*n* = 3 studies), semantic organization/categorisation (*n* = 4), mental (visual) imagery (*n* = 3), the method of loci (*n* = 3), the Preview-Question-Review-Summary-and-Test method (*n* = 1), spaced-retrieval (*n* = 1), paired association (*n* = 2), and story making (*n* = 1). Face-name association consists of pairing a picture of the face of an individual with his name. When possible, the examiner might ask the participant to elaborate on the picture in order to provide more information on the individual represented in the picture. This technique is based on mental (visual) imagery, which consists of creating a mental image of the item to remember. Mental imagery may be defined as part of the internal methods an individual uses to visually organize the information to remember [46]. Semantic organization/categorisation is a technique that is based on reorganization of the material to be learned in a way that semantically related items are grouped together and thus will have better chances to be remembered than if they were not semantically organized. The method of loci requires (1) that participants use a well-known place, like their house, in order to mentally draw a specific path. (2) Once mentally in the house, they have to choose different places or items of decoration as specific landmarks, which are later used as cues to remember the material to learn. (3) This technique also requires some mental imagery. Using mental (visual) imagery, participants must make a mental image of the item to be remembered and of the landmarks. (4) In order to retrieve the items, participants must go through their mental path to find the landmarks, and then they must retrieve the mental image they have formed during encoding [47]. Finally, the spaced retrieval technique consists of teaching participants some information that they must recall over increasing longer periods of time [48]. 

The practice of tasks in a divided attention condition was the principal intervention provided in two studies that were meant to improve attention [15, 22]. The practice of visual detection, prioritization of a task, arithmetic tasks, and speed of attention were the other techniques utilized to improve the attentional focus in 6 studies [12–15, 22, 25]. 

Tasks of monitoring, reasoning about everyday problems, problem solving, abstract reasoning, and splitting tasks in subtasks were mainly taught and practiced with participants to improve executive functioning in only 3 out of 13 studies [13, 20, 25]. The other studies did not provide training for executive functions.

### 3.6. Efficacy

All studies presented here produced, at least, one significant improvement. First, the results of between-subject comparisons will be presented followed by the results of within-subject comparisons. Most (*n* = 10/12) of the studies that performed between-subject comparisons observed an improvement in at least one of the outcome measures. However, the results of these studies (*n* = 12 studies) [12, 13, 15, 17–25] are not always clear-cut. In some studies, the group that received an intervention got a better performance [13, 19, 25] than the group who did not, but in other studies, the results depended on the outcome measure [13, 15, 17, 18, 20–25]. For instance, in Craik et al. [18], the intervention group got a better performance on the Hopkins Verbal Learning Test-Revised and on the Logical Stories Test (tests of episodic memory) but did not get better performances on other tests like the Alpha-Span [49] and Brown-Peterson [50] tests measuring working memory. The authors also mentioned that the performance of the control group improved on some outcome measures (Logical Stories Test), even if these participants did not receive any kind of intervention. This situation made it difficult to find a difference between the two groups. The authors did not explain the spontaneous improvement in the control group. However, a practice effect might at least partially account for this finding since the exact same tests were administered at the pre- and postintervention evaluations that were only 3 months apart from each other. 

 On the other hand, studies (*n* = 2) that performed only within-subject comparisons reported clearly some improvements. Belleville et al. [14] observed improvements on the face-name association measure and on the number of words recalled, but not on the measure of memory of text (i.e., Memo-text). The major reason that between-group comparisons were not performed in Belleville et al.'s [14] study is because the control group was not matched with the intervention group based on demographic features [14]. All participants of the 9 studies [12–14, 16–19, 21, 25] who received interventions targeting working memory, episodic memory and prospective memory improved significantly their performances when compared to baseline, independently of the kind of intervention and of the outcome measures. However, it should be mentioned that Buiza et al. [16] also obtained a deterioration on the working (short-term) memory measure after the training. In this study, the authors attributed this result to the normal decline in aging. They also argued that stimulation/training of short-term memory is very difficult. It is important to note that the participants in this study presented with subjective memory complaints at baseline. Unfortunately, the authors did not mention the neuropsychological tests used to include participants and to measure improvements in short-term memory. Therefore it is difficult to determine the cause of the deterioration.

Regarding attentional stimulation/training, the interventions and practice of tasks were efficient and produced significant improvements, when posttraining performances were compared to baseline performances [14, 16]. When executive functions were targeted by any kind of stimulation/training, 5 out of 6 studies demonstrated significant improvements on planning, reasoning, verbal fluency, and/or problem solving [12, 13, 16, 18, 25]. Finally, 3 studies were interested in speed of processing training [13, 14, 25]. In these two studies, the participants were asked to practice specific tasks chosen by the authors in order to improve speed of information processing. Unfortunately, the authors did not mention the characteristics of the tasks used. The results were contradictory. In the study of Ball et al. [13], in which there was a specific intervention targeting speed-of-processing, significant improvement was observed, but in the study of Belleville et al. [14], in which memory was the principal function targeted, there was no improvement. The explanation of the discrepant findings may lie in the principal objective of the respective interventions. Belleville et al. [14] did not specifically target speed of processing in the intervention they administered to participants, therefore they did not make their participants practicing as numerous tasks of speed of processing as did Ball et al. [13]. This might explain the absence of improvement on this type of activity in the study of Belleville et al.. 

Finally, Slegers et al., [23] who administered a non-specific cognitive stimulation program, observed a positive effect only on a few variables. The authors mentioned that these results were quite random and could not be directly linked to the intervention. In other words, their nonspecific intervention did not yield significant results.

## 4. Discussion

The preliminary results are promising on the tasks measuring memory, attention, executive functions, and speed of processing following the cognitive stimulation/training programs. However, the cognitive stimulation/training programs reviewed in the present study were very different from each other, had relatively small sample sizes (except for the study of Ball et al. and Willis et al. [13, 25], and usually targeted more than one cognitive function, which make conclusions regarding the efficacy of each training technique complex. For instance, some intervention programs were administered in groups with structured sessions and targeted a specific cognitive function whereas other programs were individualized or in unstructured format sessions and targeted multiple cognitive functions. 

Although the present paper reports an improvement on 1 [18] to 7 [22, 23] outcome measures following the cognitive stimulation programs, one important question still remains unanswered: the generalization of the intervention programs to everyday life activities. Only 3 studies [14, 21, 25] evaluated the generalization of the intervention program on everyday life activities of the participants, which is the key concept when the efficacy of cognitive stimulation programs must be assessed. In this paper, Belleville et al. [14] used a self-reported questionnaire that measured participant's judgement of changes in daily life [51]. They found that the training had an effect on the well-being of the participants who received the training. 

The next step is the objective evaluation of the generalization of training, by using some neuropsychological tests that will serve this purpose. In this sense, tests that have ecological validity, such as the RBMT [28] might be part of the answer, as long as they are not part of the primary outcome measures. In this sense, Mahncke et al., 2006 [21], used objective measures (i.e., RBANS) and found a generalization effect. When RBANS is not used as an outcome measure, it might be a great tool to measure a generalization effect on various cognitive domains including memory. In the future, cognitive stimulation/training studies shall administer this kind of measures.

Besides generalization of the training program to activities of daily life, another important aspect in the evaluation of the efficacy of training programs is the maintenance of the new acquired abilities. This is usually assessed using follow-up evaluations. In this paper, only 8 out of 13 studies had follow-up evaluations. In these studies, the time intervals between posttest and followups varied a lot. However, none of the studies mentioned what happened during the follow up, or even if they really knew what the participants did during this period, that is, if the participants continued to practice the tasks or not. This is an important issue that certainly should be addressed and monitored in future studies on cognitive stimulation/training programs. To this aim, future studies might, for instance, use training journals filled by the participants. 

A way to verify if the training program had an impact on the general condition of the participants might be to use a measure of quality of life. In this paper, only one study had this kind of measure. A possible explanation for this absence of measurement in the studies is that there is currently no consensus about the best tool to measure the concept of quality of life in the elderly [52]. Alternatively, the assessment of participants' self-esteem could be an interesting and appropriate variable to take into account and to assess in these programs. This could be measured using valid and sensitive self-administered questionnaires that provide information about the level of self-esteem and/or self-confidence participants had before and after the intervention. Finally, measures of instrumental activities of daily living should be added in longitudinal studies on cognitive intervention in healthy elderly to verify if cognitive training/stimulation prevents or slows down functional decline, as Willis and collaborators reported following the ACTIVE study [25].

One of the major limitations of the studies reviewed in the present paper was the use of the total score obtained on the Mini-Mental State Examination (MMSE) [53] as an exclusion criterion for individuals presenting objective memory impairment. Even if the MMSE is widely used for assessing dementia, it nevertheless presents some limitations when used with highly functioning individuals. First, participants must have severe cognitive problems to score below the cutoff that has been set for dementia. Second, the MMSE is sensitive to education and age. Third, the evaluation of episodic memory by the MMSE is very poor and lacks sensitivity for early impairments. A more exhaustive neuropsychological assessment or a more appropriate short scale to detect mild cognitive impairment, such as the MoCA [54], or at least the use of a standardized episodic memory measure would be more acceptable to characterize the level of cognitive functioning of individuals who are going to receive cognitive stimulation. In the present paper, only 4 out of 14 studies used a good and complete neuropsychological battery to assess the neuropsychological profile of their participants [12, 13, 18, 25]. Thus this aspect must definitely be improved in the future because the cognitive profile and cognitive reserve of participants included in studies evaluating efficacy of cognitive stimulation/training is one of the most important factors in the success of these programs. 

This paper did not allow the evaluation of the cost of such cognitive stimulation programs, but then it was not an objective of the present work. However, in the future, it might be an aspect worth to be assessed because it will be important for decision-makers to know the impact in terms of costs/benefits in order to offer this kind of service, if at one point this approach is deemed suitable for the healthy elderly. One might consider whom are the professionals involved in these programs and what is the amount of time devoted for such programs, by the professionals and by the participants.

Another limitation of this paper is the diversity in the training/stimulation programs. There were so many differences between the programs examined in this review that a specific prescription for an intervention cannot be given for different individuals who might want to benefit from these interventions. The next important step in order to improve understanding in this domain is to demonstrate, with rigorous experimental designs and standardized techniques of training and stimulation, what are the techniques and methods that work best to maintain and improve cognition over time. In the present demographic context, it would be important to demonstrate that such interventions could be prescribed, as much as physical activity, in order to slow down the cognitive decline observed in some elderly individuals or even to improve cognitive function. Future research will need to include much larger samples, standardized cognitive training manuals and will need to use robust experimental designs (i.e., randomized controlled trial). It will be interesting to conduct research evaluating the impact of such stimulation programs on the cognitive reserve of elderly participants and to correlate the impact of the cognitive intervention with neuroimaging data. The addition of neuroimaging data might also permit the identification of core mental processes that operate in multiple task domains, which could then be targeted by cognitive interventions in one task context and assessed for improvement in another, thus ascertaining the transfer of training [55]. 

In spite of the limits and the numerous unknown implications in the efficacy of the cognitive intervention programs, the literature demonstrates that such interventions might be efficient in patients with MCI [56–59] and even in patients presenting with mild to moderate Alzheimer's disease [60, 61]. In this sense, it is desirable to continue doing research in this domain in order to complement the pharmacological treatments currently prescribed. Finally, investigators should develop more ecological programs and compare groups of individuals involved in different cognitive activities of the daily life, such as Bridge, Sudoku, or Crosswords. Scientists and clinicians might be interested in the impacts of this kind of activities, because it is more accessible, costless and enjoyable for elderly than to be placed in an artificial laboratory context, as it was mostly done by the intervention programs reviewed in this paper. Perhaps the future of cognitive stimulation interventions relies in the activities practiced in the everyday life of the elderly.

## Figures and Tables

**Table 1:** Cognitive training in healthy elderly.

Author	Population	Age-education % women	*n*	Study design	Intervention duration (weeks) # of sessions	Time of followup	Cognitive functions targeted	Strategies	Outcome measures	Results
Auffray and Juhel [12]	Community-dwelling	A: 79.7 (8.9) E: 9.8 71%	82 64 in EC 18 in CC	pRC	N/A 6 sessions	6 & 9 months	Attention	Practice of selective and divided attention	Digit-symbol	*⇑*/b in EC & CC
							Memory	Face-name association Semantic organization	Verbal immediate and delayed recall	*⇑*/g
									Star counting task	*⇑*/g at FU-6 months
							Reasoning	Rules explication	Raven's matrices	=/b
									Verbal reasoning	=/b
									Competencies in daily life	=/b

Ball et al. [13]	Community-dwelling and senior centers	A: 73.6 (5.9) 75.9%	2832 total 711 in MT 705 in RT 712 in ST 704 in CC	RC SB	5-6 weeks 10 sessions	12 & 24 months	Memory	Semantic organization Mental imagery	Composite score from: HVLT, AVLT, and RBMT	*⇑*/g in MT
							Reasoning	Abstract reasoning Reasoning about everyday problems	Composite score from: Word series, Letter series, and Letter sets	*⇑*/g in RT
							Speed of processing	Practice under divided attention	Composite score from : UFV	*⇑*/g in ST

Belleville et al. [14]	Community-dwelling and from memory clinics for MCI	A: 66.8 (3.4) E: 13.3	47 total 9 HE in EC 20 MCI in EC 8 HE in CC 8 MCI in CC	C	8 8 sessions	No FU	Episodic memory	Mental imagery Method of loci	Face-name recall	*⇑*/b in both EC groups
								Face-name association	Unrelate word list immediate and delayed recall	*⇑*/b in both EC groups, =/b
								PQRST		
									Memo-text; short story immediate and delayed recall	=/b
							Attention	Practice of visual detection & arithmetic tasks	Brown-Peterson	=/b

Bherer et al. [15]	Community-dwelling	**HE** A: 71.0 (0.9) E: N/A 55%	88 total 32 HE in EC 12 HE in CC	RC	3 6 sessions	No FU	Attention	Priorization of a task Practice of orienting attention	RT	*⇑*/b in both EC
									Accuracy	*⇑*/g in both EC, greater for elderly
		**HA** A: 21.4 (0.9) E: N/A 59%	32 HA in EC 12 HA in CC							

Buiza et al. [16]	Community-dwelling with complaint of memory impairment	A: 74.4 (8.3) 73%	238 total 85 in EC1 68 in EC2 85 in CC	Quasiexperimental DB, stratified random	104 180 sessions	No FU	Attention/orientation	N/A	Digit span of WMS	*⇑*/b in EC1 at FU2
									Information & orientation of WMS-R	*⇑*/b in all groups at FU2
							Memory	N/A	Logic memory of WMS-R	*⇑*/b in EC1
									AVLT	
							STM	N/A	N/A	*⇑*/b in EC1, EC2 & CC ⇓/b in EC1
							Working memory	N/A	N/A	*⇑*/b in EC1
							Executive functions	N/A	Verbal fluency	*⇑*/b in EC1
							Visual motor speed	N/A	TMT-A	⇓/b in EC1, EC2 & CC
			
							Abstraction	N/A		⇓/b in EC2 & CC

Calero and Navarro [17]	Healthy elderly from retirement home	A: 76.9 (8.4) 65%	133 total 78 in EC 55 in CC	Quasiexperimental SB, C	7 14 sessions	9 months	Memory	Paired association Method of loci Categorisation	MEC	*⇑*/g at posttest & 9 months-FU
									WM	*⇑*/b in EC at posttest & 9 months-FU
							Attention spatial & temporal orientation verbal fluency		Digit-span (WAIS-III)	*⇑*/g at posttest & 9 months-FU

Craik et al. [18]	Healthy elderly with subjective complaint of cognitive memory impairment, no MCI	A: 78.7 (3.9) 55%	49 total 29 in ETG 20 in LTG	W-S crossover, BR	14 14 sessions	6 months	Memory	Psychoeducation categorisation	Alpha-Span	=/b
								Story making Mental imagery Spaced retrieval	Brown-Peterson	=/b
								Association	HVLT-R	*⇑*/g
									Logical stories	*⇑*/g and *⇑*/b in both groups

Envig et al. [19]	Community-dwelling		42 total 22 in EC 20 in CC	RC	8 8 sessions	No FU	Serial verbal recollection memory	Method of loci	Computerized test of word recognition	*⇑*/g
									Computerized test of source memory	*⇑*/g

Levine et al. [20]	Healthy elderly with subjective complaint of memory impairment, no MCI	A: 78.7 (3.9) 55%	49 total 29 in ETG 20 in LTG	W-S crossover, BR	14 14 sessions	6 months	Strategic behavior	Sequencing Splitting task in subtasks Prioritization of subgoals	SRLTs	*⇑*/g
								Monitoring	DEX	=/b in EGT & LGT

Mahncke et al. [21]	Community-dwelling	A: 70.9 E: 16.3 50%	182 total 62 in EC 61 in AC 59 in CC	RC	8–10 40–50 sessions	3 months	Speed of processing	Practice of computerized exercises	Tasks similar to exercises executed during training	*⇑*/b in EC for speed of processing & word span
							Verbal memory		RBANS	*⇑*/g (both AC & CC)

Mozolic et al. [22]	Community-dwelling	A: 69.4 E: N/A 53%	62 total 30 in EC 32 in CC	RC SB	8 8 sessions	1 month	Selective attention	Practice of exercises aiming at detecting, identifying, classifying, and sequencing visual and auditory stimuli	RT and accuracy in modality- specific tasks	RT: *⇑*/g Accuracy: *⇑*/g
									Audiovisual multisensory integration task	RT: *⇑*/g in selective visual and auditory tasks
									SDMT	*⇑*/g
									Walk and Talk paradigm	*⇑*/g
									1-Back 2-Back	*⇑*/b *⇑*/b
									Stroop-Color-Word test	=/b in both groups
									TMT	=/b in both groups
									HVLT	=/b in both groups
									POMS	=/b in both groups
									HSQ-12	=/b in both groups

Slegers et al. [23]	Community-dwelling	A: between 64 and 75 E: N/A	Total: 236 T/I: 62 T/N-I: 61 NT: 68 CC: 45	RC	2 3 sessions + personal use at home	4 & 12 months	No specific cognitive functions	Training on computer operating system, software skills, or on internet applications	VVLT_Sum 1 to 3	T/I: *⇑*/compared to T/N-I
									MCRT	=/g
									LDST	=/g
									SCWT	T/NI: *⇑* compared to T/I; NT and CC
									CFQ	=/b
									CST	T/I : *⇑* compared to T/N-I

Smith et al., [24]	Community-dwelling	A: 75.3 (6.45) E: 15.65 (2.6) 52.4%	Total: 487 EC: 242 CC: 245	RC DB	8 40 sessions	No FU	Information processing	Practice of computerized exercises	RBANS	*⇑*/g
									RAVLT	*⇑*/g
									RBMT	=/g
									WMS-III	*⇑*/g
									LNS	*⇑*/g
									Digit-span backward	*⇑*/g
									Measure of exercises performance	*⇑*/g
									CSRQ-25	*⇑*/g

Willis et al. [25]	Community-dwelling and senior centers	A: 73.6 (5.9) 75.9%	2832 total 711 in MT 705 in RT 712 in ST 704 in CC	RC SB	5-6 weeks 10 sessions	60 months	Memory	Semantic organization Mental imagery	Composite score from: HVLT, AVLT, and RBMT	*⇑*/g in MT
							Reasoning	Abstract reasoning Reasoning about everyday problems	Composite score from: Word series, Letter series Letter sets	*⇑*/g in RT
							Speed of processing	Practice under divided attention	Composite score from : UFV	*⇑*/g in ST
									MDS-HC	*⇑*/g for RT
									EPT	=/g
									OTDL	=/g
	

## Abbreviations

*⇑*/b: Improvement compared to baseline performance
*⇑*/g: Improvement compared to control group performance
=/b: no difference compared to baseline performance
=/g: no difference compared to control group performance
A (age): mean years
AC: Active control group
AVLT: Auditory Verbal Learning Test
BNT: Boston Naming Test
BDAE: Boston Diagnostic Aphasia Examination
BR: Block-randomized
C: Controlled
CC: Control condition
CDT: Clock Drawing Test
CFQ: Cognitive failure Questionnaire
CS: Cognitive stimulation
CSRQ: Cognitive Self-Report Questionnaire
CST: Concept Shifting Test
CT: Cognitive training
DB: Double-blind
DEX: Dysexecutive questionnaires
E: Elderly
EC: experimental condition
EG: Everyday group
EPT: Everyday Problems Test
ETG: Early Training group
FU: followup
FU1: followup after 1 year
FU2: followup after 2 years
HA: Healthy adults
HE: Healthy elderly
HSQ: 12-Item Health Status Questionnaire
HVLT: Hopkins Verbal Learning Test
HVLT-R: Hopkins Verbal Learning Test—Revised;
LDST: Letter-Digit Substitution Test
LG: Laboratory group
LNS: Letter-Number-Sequencing
LTG: Late Training Group
MEC: Mini Examen Cognoscitivo (Spanish version of MMSE)
MCRT: Motor Choice Reaction Time test
MDS-HC: Minimum Data Set Home Care
MS: Motor sequences of Luria
MT: Memory training
N/A: Not mentioned
NR: Nonrandomized study
NT: No training
OE: Old elderly
OTDL: Observed Tasks of Daily Living
POMS: Profile of Mood States
pRC: Pseudo random controlled study
PROMS: Prospective Memory Screening Test
RAVLT: Rey Auditory Verbal Learning Test
Random: randomisation
Psychoed: Psychoeducation
RBANS: Repeatable Battery for the Assessment of Neuropsychological Status
RBMT: Rivermead Behavioral Memory Test
RC: Randomized-controlled study
RT: Reasoning training
SB: Single-blind
SCWT: Stroop Color Word Test
SDMT: Symbol-Digit Modality Test
SRLTs: Simulated real life tasks
ST: Speed training
T/I: Training-intervention
TMT: Trail Making Test
T/N-I: Training-no intervention
UFV: Useful Field of View (tasks 2–4)
VVLT: Visual Verbal Learning Test
WAIS-III: Weschler Adult Intelligence Scale-III
WM: Working memory
WMS: Weschler Memory Scale
WMS-R: Weschler Memory Scale-Revised
WMET: Working Memory Evaluation Test
W-S: Within-subjects
YA: Young adults
YE: Young elderly.
